# Effects of extremely low frequency electromagnetic fields on paraoxonase serum activity and lipid peroxidation metabolites in rat

**DOI:** 10.1186/s40200-014-0085-2

**Published:** 2014-08-13

**Authors:** Soroush Seifirad, Shahrokh Farzampour, Mitra Nourbakhsh, Mahsa Mohammad Amoli, Maryam Razzaghy-Azar, Bagher Larijani

**Affiliations:** Endocrinology and Metabolism Research Center, Endocrinology and Metabolism Clinical Sciences Institute, Tehran University of Medical Sciences, Tehran, Iran; Electromagnetic Waves Research Center, Artesh University of Medical Sciences, Tehran, Iran; Metabolic Disorders Research Center, Endocrinology and Metabolism Molecular Sciences Institute, Tehran University of Medical Sciences, Tehran, Iran

**Keywords:** Paraoxonase, Lipid peroxidation, Electromagnetic radiation, Magnetic fields, Free fatty acids

## Abstract

**Background:**

Atherogenic effects of ELF-MF exposure have not been studied well so far. Therefore we have hypothesized that ELF-MF exposure might have atherogenic effect by impairing antioxidant function and increasing lipid peroxidation. This study was therefore undertaken to examine the effects of ELF-MF on paraoxonase (PON) activity, antioxidant capacity and lipid peroxidation metabolites. Effects of time on remodeling of antioxidant system were also investigated in this study.

**Methods:**

Seventy five Wistar rats were randomly allocated into five groups as follows: 1) Sham exposure, 2) Single exposure to 60 Hz, sacrificed immediately after exposure, 3) Single exposure to 60 Hz, sacrificed 72 hours after exposure, 4) Fourteen days of exposure to 60 Hz, sacrificed immediately after exposure, and 5) Fourteen days of exposure to 60 Hz, sacrificed 72 hours after exposure. Blood samples were collected and analyzed. The results were compared using ANOVA and post hoc Tukey HSD for multiple caparisons.

**Results:**

Single ELF-MF exposure significantly increased lipid peroxidation (CD and MDA) and increased antioxidant serum activity (HDL, paraoxonase activity, and serum total antioxidant capacity). Chronic ELF-MF exposure increased lipid peroxidation and affected antioxidant system. Free fatty acids levels were significantly increased after both single and two weeks exposure. Chronic exposure led to irreversible changes while acute exposure tended to reversible alterations on above mentioned parameters.

**Conclusions:**

According to the results of this study, ELF-MF exposure could impair oxidant-antioxidant function and might increase oxidative stress and lipid peroxidation. Antioxidant capability was dependent on the duration and continuity of ELF-MF exposure.

## Introduction

Exposure to extremely low frequency magnetic fields (ELF-MF) is increased due to modernization and development of new electronic devices. Several adverse effects such as psychological and carcinogenic effects were reported as a result of exposure to extremely low frequency magnetic fields (ELF-MF) [1,2]. However atherogenic effects of ELF-MF exposure have not been studied well. It is hypothesized that ELF-MF exposure might impair antioxidant systems and increase lipid peroxidation [3-5].

Low density lipoprotein (LDL) oxidation in arterial walls was suggested as an important mechanism of atherosclerosis. On the other hand, antioxidants, high density lipoprotein (HDL) and paraoxonase (PON) activity are among the main protective factors of lipid peroxidation and therefore atherosclerosis. It has been shown that PON decreases oxidation sensitivity of LDL [6,7].

To the best of our knowledge, there is no report for investigating the effects of ELF-MF on PON activity. Also the results of previous studies on effects of ELF-MF on lipid peroxidation are still contradictory [3,4,8,9]. This study was therefore undertaken to examine the effects of ELF-MF on PON activity, antioxidant capacity and lipid peroxidation metabolites. Effects of time on remodeling of antioxidant system were also investigated in this study.

## Materials and methods

### Animals

Seventy five 220–250 g adult male Wistar rats were included in the study. In order to adapt with temperature, humidity, and regular light (12 hr)/dark (12 hr) cycle, the animals were stored for one week in the animal laboratory before ELF-MF exposure. The temperature was set at 23°C and normal food (AIN-93 purified diets for laboratory rodents) and water was always available in the animals’ cage.

### ELF-MF exposure

Using a modified form of a previously described method, ELF-MF exposure was set up [10]. We tried to produce 60 Hz frequency and 0.5 mT field using a pair of Helmholtz coils which were set with winding surrounded in a plexi-glass frame. Through a variable transformer the coils were connected to a 220 V AC power supply. The centrally horizontal magnetic field was homogenous in a plexi-glass cage (a non-magnetic material). Sham exposure group were placed in the same cage though the coils were turned off, being exposed to the local geomagnetic field (BH = 0.18, BV = 0.22, BT = 0.23) and BAC < 0.02 G). By means of a gauss-meter (Lake Shore, Model 410), the homogeneity in the plexi-glass was determined. To adjust the frequency and intensity, the coils were linked to a waveform generator.

### Methodology and serum analysis

Seventy five Wistar rats were randomly allocated into five groups as follows:

1. Sham exposure
2. Single exposure to 60 Hz, sacrificed immediately after exposure
3. Single exposure to 60 Hz, sacrificed 72 hours after exposure
4. Fourteen days of Exposure to 60 Hz, sacrificed immediately after exposure
5. Fourteen days of Exposure to 60 Hz, sacrificed 72 hours after exposure

The rats were sacrificed after anesthesia with diethyl ether; blood samples were then collected through left ventricle after thoracotomy.

Serum levels of HDL and LDL were measured using an automated chemical analyzer (Abbott analyzer).

Paraoxonase activity was determined spectrophotometrically using phenylacetate (substrate) and paraoxone (O, O-diethyl-O-P-nitrophenylphosphate) as described previously [11]. Malondehaldeid (MDA) levels were measured using thiobarbituric acid (TBA) method based on previous method [12]. Serum total antioxidant capacity (TA) was measured spectrophotometerically [TAS kit (“Total Antioxidant Status” – TAS; Randox, Crumlin, UK)].

Enzymatic colorimetric methods (Spectrophotometery) were used to assess free fatty acids (FFA) levels in blood samples (not heparinized, centrifuged as soon as possible and frizzed).

Conjugated diens (CD) levels were also measured spectrophotometrically (wavelength range 230–235) [13,14].

### Statistical analysis

Kolmogorov-Smirnov test was used to check for the normality of data distribution. One way ANOVA test was used to compare HDL, LDL, TA, PON, MDA, FFA, and CD between groups. For significant variables post hoc Tukey HSD test was applied to compare between groups. For five possible comparisons the p value was set as 0.01 using Bonferroni correction (0.05/5).

## Results

**Figure 1:**
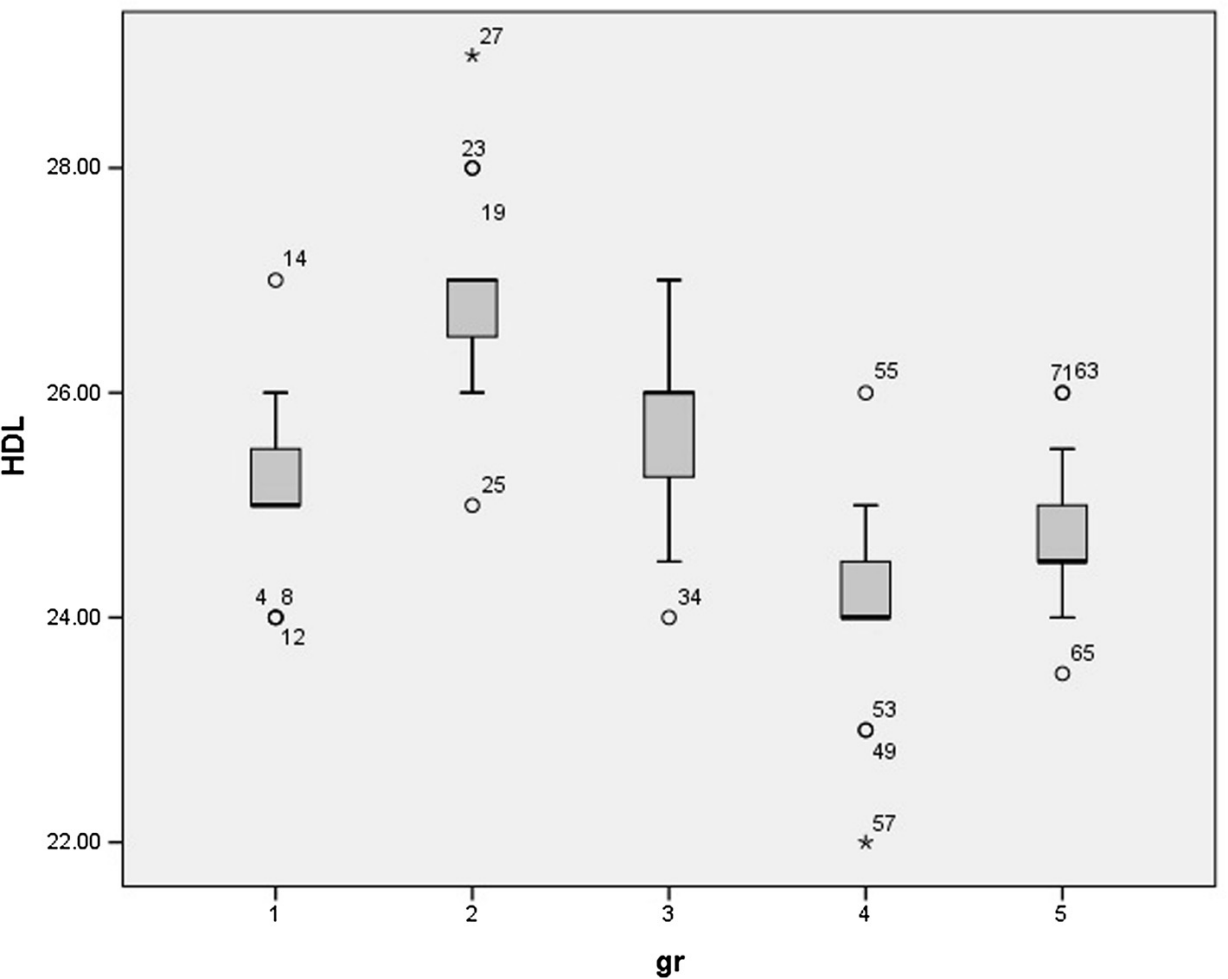
**Serum levels of high density lipoprotein (HDL) in all studied groups.**

**Figure 2:**
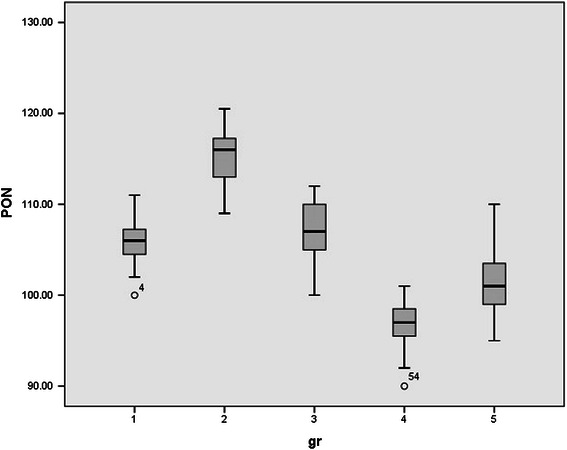
**Serum paraoxonase (PON) activity in all studied groups.**

**Figure 3:**
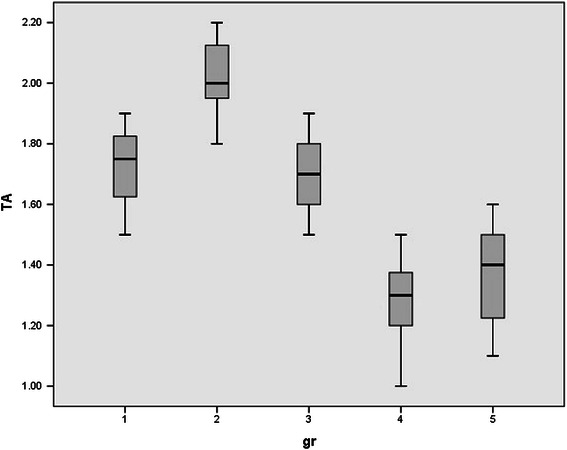
**Serum levels of total antioxidant (TA) in all studied groups.**

**Figure 4:**
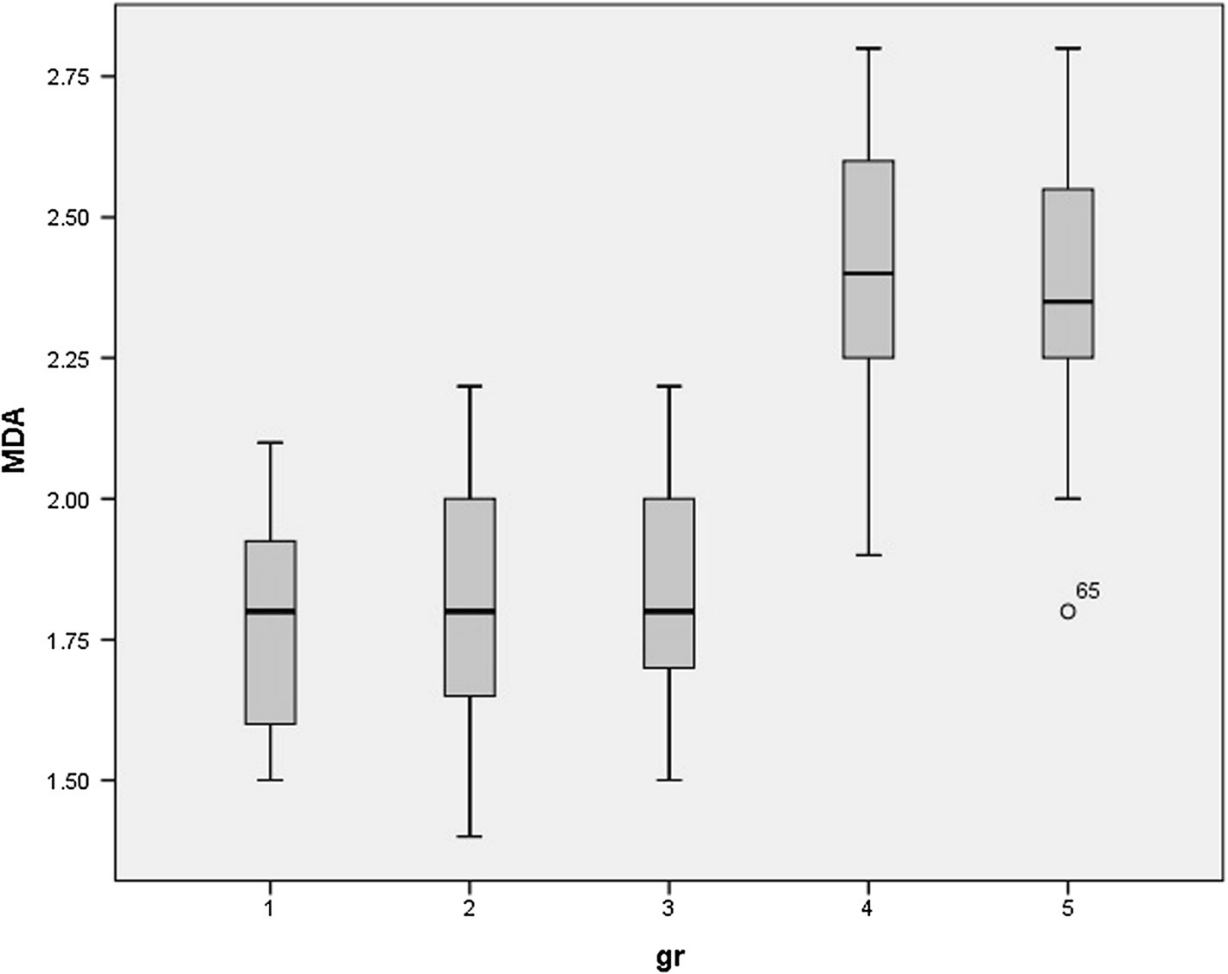
**Serum levels of malondehaldeid (MDA) in all studied groups.**

**Figure 5:**
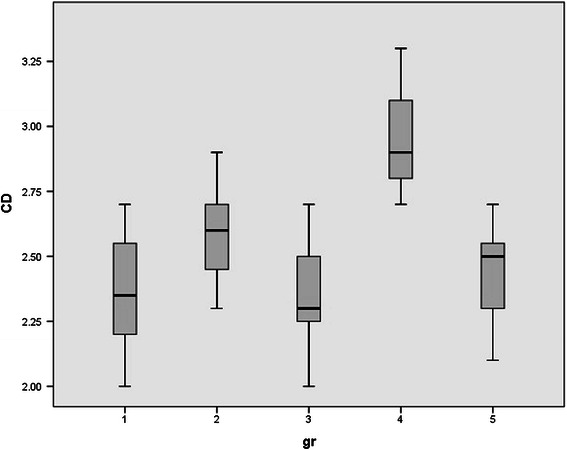
**Serum levels of conjugated diens (CD) in all studied groups.**

**Figure 6:**
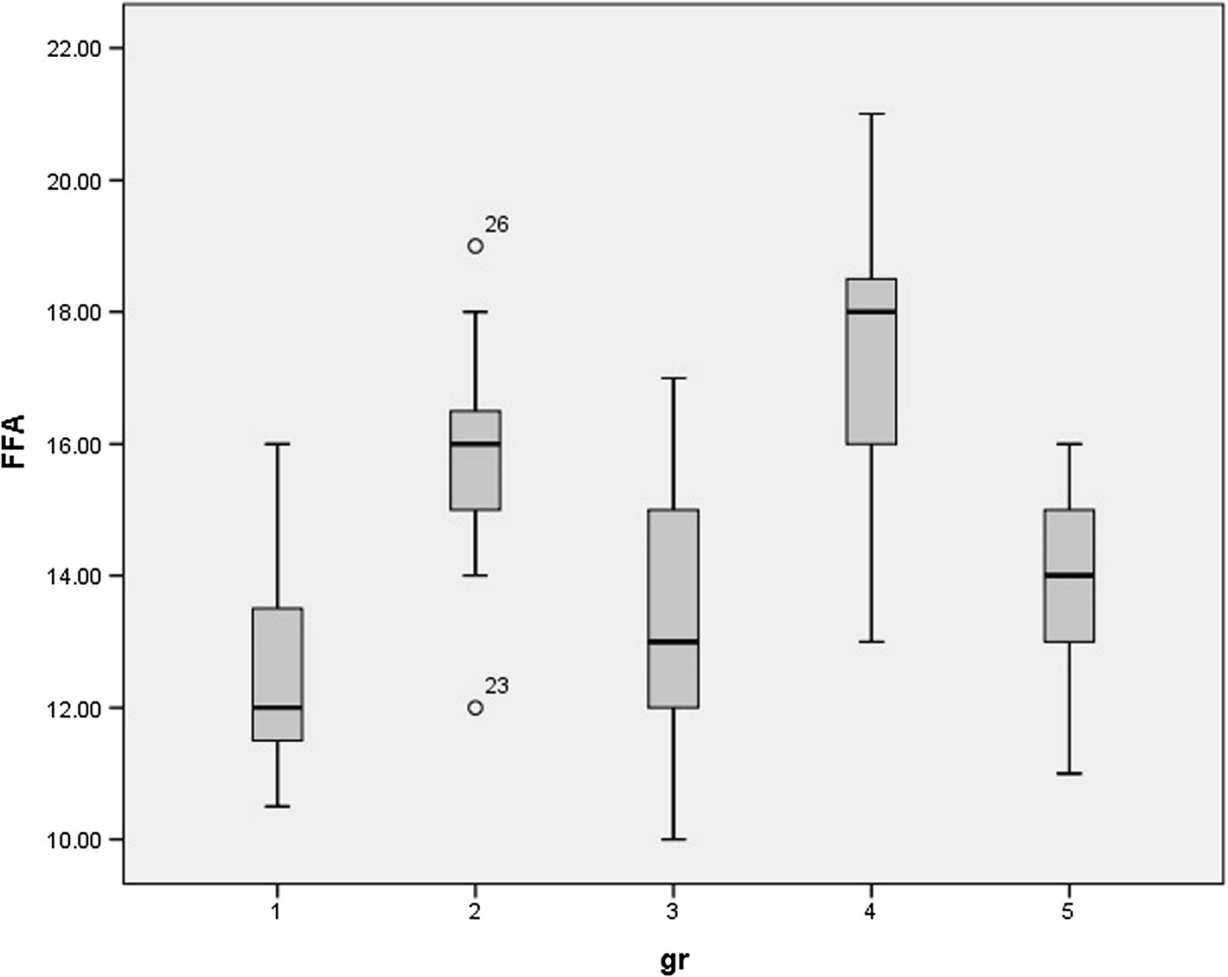
**Serum levels of free fatty acids (FFA) in all studied groups.**

**Figure 7:**
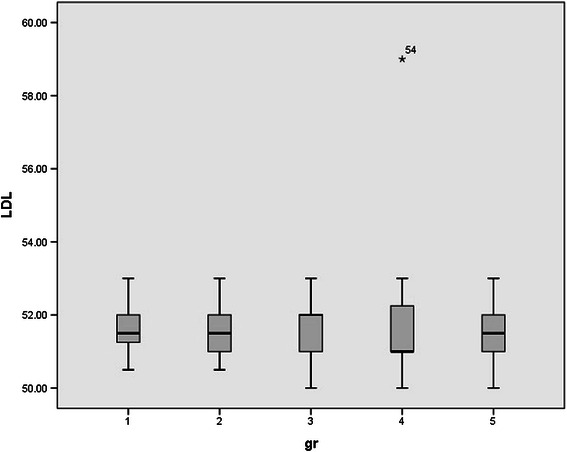
**Serum levels of low density lipoprotein (LDL) in all studied groups.**

Since rats were randomly allocated in five groups, no significant differences were observed between the mean weights of rats. (p value > 0.05). Normal distribution of data was observed between groups. Serum levels of HDL, LDL, FFA, TA, CD, MDA, and serum PON1 activity in all studied groups were shown in Table 1 and Figures 1,2,3,4,5,6,7.

**Table 1:** **Serum levels of HDL, LDL, FFA, TA, CD, MDA, and serum PON1 activity at the end of the study in all studied groups**

**Parameter**	**Sham exposed**	**4 hrs-suddenly**	**4 hrs-after 72 hrs**	**2 W-suddenly**	**2 W-after 72 hrs**
**PON1 (U/mL)**	105.90 ± 2.91^*^	115.20 ± 3.50	106.90 ± 3.93	96.76 ± 2.89	101.43 ± 3.79
**MDA (nmol/mL)**	1.77 ± 0.21	1.81 ± 0.23	1.84 ± 0.22	2.40 ± 0.25	2.36 ± 0.26
**CD (mg/dL)**	2.37 ± 0.21	2.58 ± 0.19	2.35 ± 0.20	2.95 ± 0.18	2.42 ± 0.18
**HDL (mg/dL)**	25.13 ± 0.83	26.93 ± 96	25.70 ± 0.84	24.06 ± 0.96	24.73 ± 0.67
**LDL (mg/dL)**	51.70 ± 0.70	51.66 ± 0.77	51.70 ± 0.81	51.93 ± 2.12	51.60 ± 0.82
**FFA (mg/dL)**	12.60 ± 1.58	15.80 ± 1.78	13.53 ± 1.99	17.26 ± 2.18	13.66 ± 1.59
**TA (mg/dL)**	1.72 ± 0.13	2.03 ± 0.12	1.17 ± 0.11	1.28 ± 13	1.36 ± 0.15

*Mean ± Standard deviation.

We observed a significant increase in the serum levels of CD in one time exposed rats (4 hrs, 60 Hz) compared to the sham exposed group (p value < 0.000). After 72 hours, serum CD levels were decreased and normalized (p value > 0.05).

A similar pattern was observed in chronic exposed rats, however the increased CD levels were more remarkable compared to one time exposed rats (p value < 0.000).

Serum levels of MDA, as a late marker for peroxidation, were also measured to assess the effect of ELF-MF exposure on lipid peroxidation. At the end of the study, MDA levels in one time exposed rats were slightly higher compared to the control group but the observed difference was not statistically significant (p value = 0.990). In chronic ELF-MF exposed group, serum MDA levels were even higher than the one time exposed group and were remained high after three days (p value <0.000).

No statistically significant difference in the concentration of LDL was observed between sham exposed and one time exposed rats at the end of study. (p value = 0.998) serum LDL levels were not changed after chronic ELF-MF exposure (p value = 0.983).

However, FFA serum levels were increased after either acute (one time) (p value <0.000) or chronic (2 weeks) (p value < 0.000) ELF-MF exposure, which demonstrated increased lipid metabolism after ELF-MF exposure. After 72 hours, serum levels of FFA were normalized. There were no significant difference between the serum FFA levels of acute (p value = 0.638) and chronic (p value = 0.511) ELF-MF exposed rats after 72 hours compared to the sham exposed rats.

Serum HDL levels were increased after one time exposure (p value <0.000) while chronic exposure to ELF-MF decreased HDL serum concentrations. (p value = 0.010) Seventy two hours after one time exposure, serum HDL levels were normalized, (p value = 0.381) however 3 days after chronic exposure, decreased HDL levels were not compensated and HDL serum levels were remained low (Slightly increased but remained in the same subset with suddenly sacrificed rats).

Parallel to the HDL changes pattern, serum paraoxonase activity increased and decreased after acute (p value < 0.000) and chronic (p value < 0.000) ELF-MF exposure respectively. After 72 hours paraoxonase serum levels were normal in one time exposed rats, (p value = 0.931) while its serum levels remained low in two weeks ELF MF exposed rats (p value = 0.006).

Serum TA levels were increased after one time ELF MF exposure. On the other hand, in chronic exposed rats TA levels were decreased significantly. Three days after single exposure serum TA levels were normalized, (p value = 0.997) while in chronic exposed rats serum TA levels were remind low after 72 hours compared to the sham exposed group (p value < 0.000).

## Discussion

This study was undertaken to evaluate effects of acute and chronic ELF-MF exposure on serum lipid profile, lipid peroxidation, antioxidant system, and paraoxonase activity as mediators protecting against atherogenesis and atherosclerosis.

According to the results of our study, ELF-MF exposure changed lipid profile, increased lipid peroxidation, and affected antioxidant system.

Paraoxonase, HDL and serum total antioxidant capacity parallel alterations reflects their similar functions as measurements of antioxidant system. In this study, one time acute exposure to ELF-MF slightly increased their serum levels which showed that antioxidant system has been evoked. These mediators levels were normalized after 3 days in short time exposure, however after chronic exposure, antioxidant capacity remained low after 72 hours. Antioxidant capacity could be repaired after exposure, but its’ ability to repair is dependent on duration and continuity of ELF-MF exposure. It should be noted that observed differences in HDL levels were not clinically significant; clinical significance of the above statistically observed differences needs further evaluation.

To the best of our knowledge, this is the first study evaluating the paraoxonase alterations after acute and chronic ELF-MF exposure. According to the results of Torres-Duran et al. serum HDL levels were increased after acute one time exposure to ELF- MF (Hz). Their results were in accordance with our findings. [15] It has been shown that paraoxonase has antioxidant properties and could prevent cell-mediated oxidative modification of low density lipoprotein [7]; hence, impaired paraoxonase activity could increase risk of atherosclerosis due to increased lipoprotein peroxidation [6].

LDL seems to be the impervious particle in this study. No significant differences were observed between LDL levels of different groups. Although increased levels of free fatty acids in our study might indicate that metabolism of lipids were increased after ELF-MF exposure. Most previous reports suggest that ELF-MF exposure might increase lipid metabolism [15]. Torres-Duran et al. observed that one time exposure to ELF-MF significantly decreased total cholesterol levels and increased lipid peroxide content in the liver. Serum free fatty acids level were increased in their exposed rats compared to their control sham exposed rats [15]. Nitric oxide synthase stimulation by ELF-MF may explain these findings. CD and MDA levels were increased after ELF-MF exposure in our study. Surprisingly, even one time exposure to ELF-MF increased CD levels as an early stage marker of lipid peroxidation. While after 72 hours, CD levels were normalized but MDA levels increased as the late stage marker of peroxidation. Chronic exposure tends to increased levels of CD and MDA. Due to the kinetic of CD particle, its serum levels were normalized after 72 hours but MDA serum levels remained high. This was predictable due to short half life of CD particles as early markers of lipid peroxidation. MDA prolonged half life explains observed steady state of MDA levels after 72 hours.

According to the study by Zwirska-Korczala et al., ELF-MF exposure increased lipid peroxidation in cultured 3 T3-L1 pre-adipocyte [3]. Erdal et al. suggested that ELFMF exposure could increase nitrosative-oxidative stress in liver tissue [16]. Ayata et al. showed that Exposure to mobile phone waves increased lipid peroxidation and fibrosis in Rat skin [4]. Lee et al. suggested that ELF-MF exposure affects antioxidant system by production of free reactive oxygen species ROS [10]. Atherosclerosis is a chronic process, and chronic lipid peroxidation by ELF-MF exposure theoretically might increase the risk of atherosclerosis.

It is well known that inflammation and oxidative stress are closely linked while oxidative stress evokes inflammation; on the other hand inflammation increases oxidative stress via production of reactive oxygen species through inflammatory cells. In a recent published study by Jonai et al. ELF-MF exposure decreased TNF-α production in the human peripheral blood mononuclear cells (h-PBMCs). Interleukin 1-B production was increased and Interferon-γ production was decreased at some point of exposure [17]. IL-1 is an inflammatory stimulating cytokine; increased production of IL-1 could increase oxidative stress. Surprisingly, there are some reports suggesting ELF-MF as anti-inflammatory mediator in treatment of inflammatory conditions [18,19]. However as we mentioned above, most of recent published articles are in line with the results obtained in our study implying ELF-MF exposure as mediator of oxidative stress [4,10,16,20]. Akdag et al. showed that ELF-MF exposure (100 and 500 micro T) implemented oxidative stress, diminished antioxidant defense system and had toxic effects on brain of exposed rats [20]. According to the results of a recent published study by Jelenkovic et al., ELF-MF exposure increased lipid peroxidation in frontal cortex, forebrain and basal areas in the brain of exposed rats. Superoxide anion production was also increased in all areas of brain in exposed rats. They suggested that NO2 signaling pathways might be affected by NO2 reaction with superoxide onions [5].

According to the results of our study, one time ELF-MF exposure increased lipid peroxidation (CD and MDA) and increased antioxidant serum activity (HDL, paraoxonase activity, and serum total antioxidant capacity). Chronic ELF-MF exposure increased lipid peroxidation and affected antioxidant system. Free fatty acids levels were increased after both one time and two weeks exposure. Chronic exposure made irreversible changes while acute exposure tended to reversible alterations on above mentioned parameters. It seems that ELF-MF exposure implements an effect which evokes antioxidant system to recompense toxic effects of reactive oxygen species; however, in chronic ELF-MF exposure antioxidant system is exhausted and oxidative stress and resulted lipid peroxidation is dramatically increased as a consequence.

Glutathion peroxidase levels as a valuable marker of oxidative stress were not measured in this study. Clinical significance of observed changes in measured parameters needs further evaluations.

Oxidant-antioxidant pathways are extremely complex and it should keep in mind that uncontrolled and over the counter consumption of any supplement could accompany by several unfavorable and hazardous effects [21]. Manipulation and modification of oxidant-antioxidants pathway needs accurate knowledge of this complex system. Hence, our findings and even the results of larger clinical studies do not warranty benefits of antioxidants consumption.

In conclusion, ELF- MF exposure could impair oxidant-antioxidant balance and might increase oxidative stress and lipid peroxidation. Antioxidant defect could be repaired after exposure; however it might depends on the duration and continuity of ELF-MF exposure.

### Ethical standards

All performed experiments and procedures were in accordance with the guidelines for animal care and use of ethics committee of Tehran University of Medical Sciences and ethical standards laid down in the 1964 Declaration of Helsinki and its later amendments. The study protocol was approved by the ethics committee of Endocrinology and Metabolism Clinical Sciences Institute, Tehran University of Medical Sciences.
